# The Flip Tilt Illusion: Visible in Peripheral Vision as Predicted by the Central-Peripheral Dichotomy

**DOI:** 10.1177/2041669520938408

**Published:** 2020-07-30

**Authors:** Li Zhaoping

**Affiliations:** Max Planck Institute for Biological Cybernetics, University of Tübingen

**Keywords:** illusion, perception, neural mechanisms, top-down feedback, attention, peripheral vision, primary visual cortex (V1), feedforward-feedback-verify-(re)weight, central-peripheral dichotomy, analysis-by-synthesis

## Abstract

Consider a gray field comprising pairs of vertically aligned dots; in each pair, one dot is white the other black. When viewed in a peripheral visual field, these pairs appear horizontally aligned. By the Central-Peripheral Dichotomy, this flip tilt illusion arises because top-down feedback from higher to lower visual cortical areas is too weak or absent in the periphery to veto confounded feedforward signals from the primary visual cortex (V1). The white and black dots in each pair activate, respectively, on and off subfields of V1 neural receptive fields. However, the sub-fields’ orientations, and the preferred orientations, of the most activated neurons are orthogonal to the dot alignment. Hence, V1 reports the flip tilt to higher visual areas. Top-down feedback vetoes such misleading reports, but only in the central visual field.

Figure 1A to F comprises homo-pairs and hetero-pairs of dots of the same and opposite contrast polarities, respectively. Eight adults casually viewed 40 such images, each contained only homo- or hetero-pairs which were all vertically (as in Figure 1A and B) or all horizontally aligned. Each image was shown for about 200 to 500 milliseconds, extended around 5°–10° in visual angle, and was at 5°–15° eccentricity when viewed peripherally. Observers reported the alignment confidently and correctly when the images were viewed centrally. When the images were viewed peripherally, the reports for the homo-pair and hetero-par images were almost 100% correct and 100% *incorrect*, respectively, and were unconfident, especially for the hetero-pair images.

**Figure 1.**
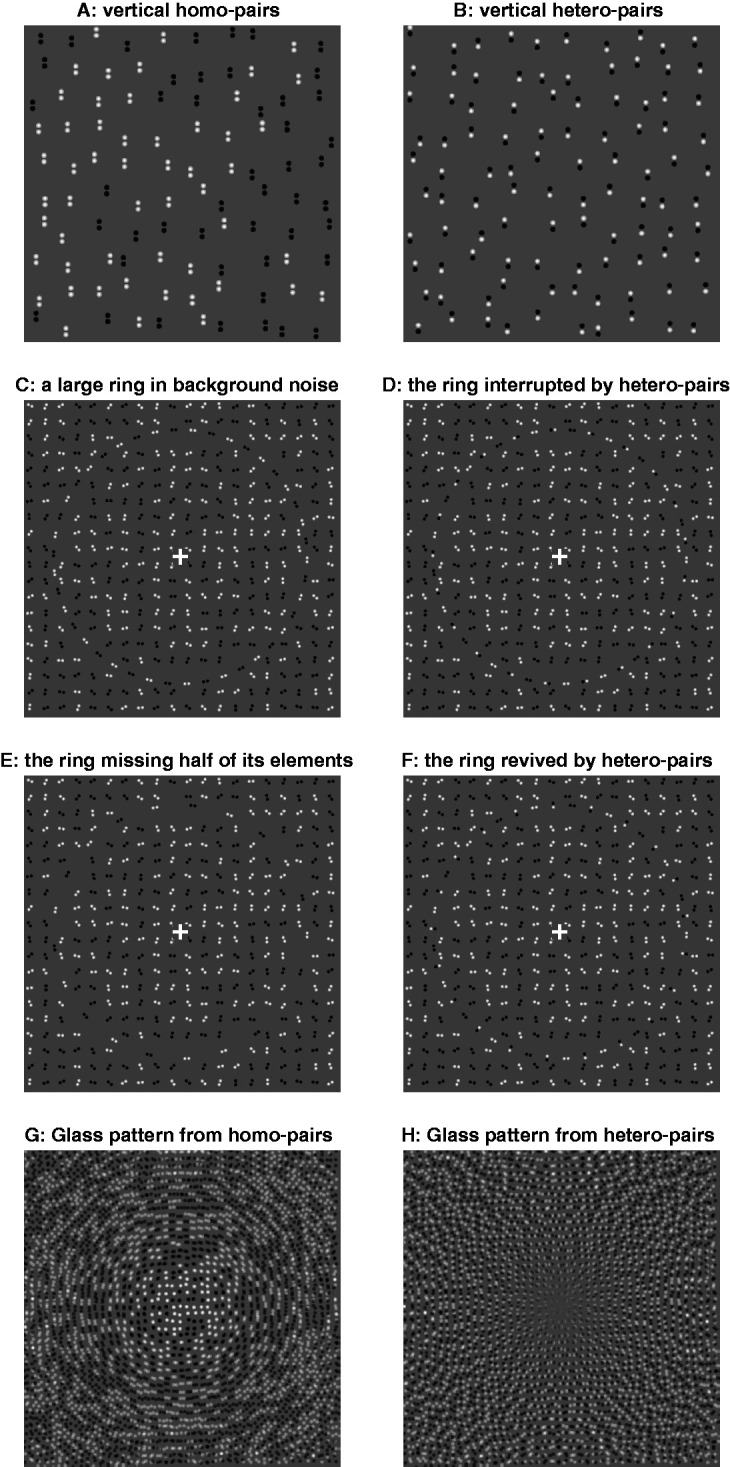
Vertical homo- (A) or hetero-pairs (B). Viewed peripherally, the pairs in (B) appear horizontal. C–F: Fixate the central cross, the large ring (in background noise) is more noticeable in (C) and (F) than (D) and (E). Only the elements of the rings differ. (G) and (H): Glass patterns by superposing a field of black and white dots and its slightly (by $2^{o}$) rotated (and contrast-inverted for H) version.

The illusion is clearer in Figure 1C to F: A large ring formed from homo-pairs parallel to its tangent (Figure 1C) perceptually weakens when every second homo-pair is removed (Figure 1E) or replaced by a parallel hetero-pair (Figure 1D), but remains perceptually strong when the hetero-pairs are orthogonal to the tangent (Figure 1F).

This illusion likely explains the change from circular to a more radial appearance of Glass patterns (Glass, 1969) when homo-paired dots (from image rotation and superposition, Figure 1G) become hetero-paired ones (Figure 1H, Stuart Anstis, private communication, May 4, 2010).

A vertical hetero-pair could excite a horizontally-tuned primary visual cortex (V1) neuron (Figure 2B), when each dot is in the contrast-corresponding subfield of its visual receptive field (RF), but is ineffective to excite a vertically-tuned V1 neuron (Figure 2C). Meanwhile, these neurons are also activated by homo-pairs whose alignment matches their preferred orientation. Thus, a V1 neuron’s feedforward signal to higher visual areas provides multiple alternative hypotheses regarding which retinal input, for example, for Figure 2B, the vertical hetero-pair, one of the two horizontal homo-pairs, or even a horizontal Gabor, actually causes this signal (Figure 3). Such an ambiguity arises if V1 does not send forward all visual input information available in V1. That attentional selection, which massively culls information along the visual pathway, starts at V1’s output is argued by Zhaoping (2019), motivated by a bottom-up saliency map created by V1 (Li, 2002) to guide gaze shifts. For example, information in V1 about the eye of origin of visual inputs and many spatial details are absent in downstream areas.

**Figure 2.**
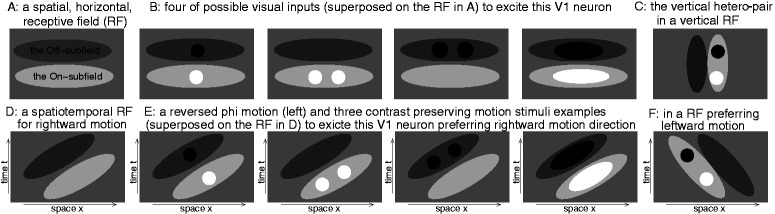
A: Schematic of the Gabor-like, horizontally oriented, spatial receptive field (RF) of a V1 neuron, with on- and off-subfields. B: Activation of (A) neuron by a vertical hetero-pair; horizontal homo-pairs and a horizontal Gabor. C: Vertical hetero-pair in a vertical RF. D to F: As in (A) to (C) but for spatiotemporal RFs (space/time along the horizontal/vertical axis) and motion stimuli.

**Figure 3.**
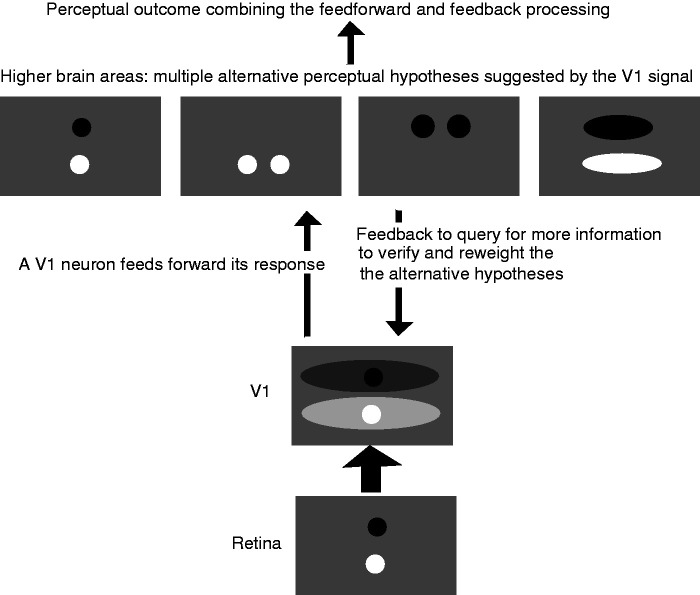
Feedforward-Feedback-Verify-and-(Re)Weight (FFVW) Process of Perception. The attentional bottleneck prevents feedforward V1 signals from conveying fully detailed retinal input information available in V1, making it ambiguous which of the multiple alternatives is responsible for the feedforward V1 signals. This ambiguity is resolved when feedback queries for specific additional information to narrow down among the alternatives to reach a final perceptual outcome.

When needed to resolve the ambiguity caused by feedforward information loss, top-down feedback queries V1 for additional information, using a form of analysis-by-synthesis (Zhaoping, 2017) to see whether the would-be visual input for each alternative hypothesis (suggested by the feedforward V1 signals) matches the actual inputs in V1. The best-matching hypothesis is favored in the final percept. This process is termed feedforward-feedback-verify-and-(re)weight (see the review in Zhaoping, 2019). Motivated by behavioral data, Zhaoping (2017) further proposed the Central-Peripheral Dichotomy (CPD): that this top-down feedback is weaker or absent in peripheral vision. Accordingly, misleading feedforward inputs from V1 cannot be as easily verified or vetoed peripherally by feedback. In particular, a hetero-pair as in Figure 3 would be perceived as horizontally oriented, as suggested by the majority of the hypotheses consistent with the feedforward V1 signal.

This illusion is analogous to reversed phi motion (Anstis, 1970): the perceived direction of motion of two sequentially presented, spatially displaced, images of opposite contrast polarities is opposite to the spatial displacement (Figure 2D to F). This visual input activates motion-direction-tuned V1 neurons (Figure 2D to F) exactly analogous to that in Figure 2A to C, given the orientation of their RFs in space-time rather than just space. Equally, the flip tilt illusion is also analogous to the reversed depth illusion (Zhaoping & Ackermann, 2018), where presentation of opposite polarity images is to the two eyes rather than at two times. Consistent with the CPD, reversed phi motion and reversed depth perception (the latter being another original prediction of CPD) is stronger, or only visible, peripherally.

Further, one can ask, for example, whether the illusory movement of black-and-white dotted lines in Ito et al. (2009), also stronger in the periphery, is related to the flip tilt illusion, whether second- or higher-order visual processes, which operate nonlinearly on image pixel values and more in central vision, are due to the top-down feedback, and whether very brief viewings to prevent effective feedback (as in Zhaoping, 2017) could evoke flip tilt illusion in central vision (also pointed out by Stuart Anstis).

More generally, Zhaoping (2019) suggested that assessing the relative strength of a visual illusion or phenomenon in central versus peripheral fields could determine whether feedback or feedforward mechanisms are mainly responsible. This tests the CPD, and helps the vast collection of visual illusions contribute rather specifically to investigations of the neural mechanisms of vision.
